# Impact of Latent Tuberculosis on Severity and Outcomes in Admitted COVID-19 Patients

**DOI:** 10.7759/cureus.19882

**Published:** 2021-11-25

**Authors:** Manu Madan, Bhvya Baldwa, Arun Raja, Rahul Tyagi, Tanima Dwivedi, Anant Mohan, Saurabh Mittal, Karan Madan, Vijay Hadda, Pawan Tiwari, Randeep Guleria

**Affiliations:** 1 Pulmonary Critical Care and Sleep Medicine, All India Institute of Medical Sciences, New Delhi, New Delhi, IND; 2 Laboratory Oncology, All India Institute of Medical Sciences, National Cancer Institute, Jhajjhar, Jhajjhar, IND

**Keywords:** covid-19, covid-19 severity, ltbi, mantoux test, latent tuberculosis

## Abstract

Background

There is limited data on coronavirus disease 2019 (COVID-19) and latent tuberculosis infection (LTBI).

Methodology

We analyzed data of admitted COVID-19 patients evaluated for LTBI to examine the impact of LTBI on severity, laboratory parameters, and COVID-19 outcome. Prospectively collected data were analyzed for 60 patients who were administered the Mantoux tuberculosis skin test (TST) using five tuberculin units of purified protein derivative. All patients were administered TST irrespective of Bacille Calmette-Guérin (BCG) vaccination status. Comorbidities, clinical features, radiologic involvement, laboratory parameters, and clinical course were analyzed concerning LTBI.

Results

The mean age was 45.9 (±15.2) years, and 35 (58.3%) patients had non-severe disease. The vast majority (n = 56/60; 93.3%) had been vaccinated with single-dose BCG in infancy or early childhood, as per national immunization guidelines. LTBI was diagnosed in 15 (25%) patients. LTBI prevalence was lower in severe (n = 1/25; 4%) than non-severe (n = 14/35; 40%) COVID-19 (p = 0.01) patients. LTBI patients had lower percentage neutrophil count, higher lymphocyte percentage, higher monocyte count, lower neutrophil-lymphocyte (NL) ratio, lower alanine aminotransferase, lower C-reactive protein, and lesser radiologic involvement compared to those without LTBI (p < 0.05). Similarly, among the mild COVID-19 subgroup, those with LTBI had higher lymphocyte and monocyte counts and lesser radiologic involvement than those without LTBI (p < 0.05).

Conclusions

LTBI patients appear to have milder disease, higher lymphocyte and monocyte count, higher NL ratio, and lesser radiographic involvement. This observation needs to be studied in larger studies using interferon release assays.

## Introduction

Coronavirus disease 2019 (COVID-19) is an infection caused by a novel coronavirus, termed severe acute respiratory syndrome coronavirus 2 [1]. First identified in late 2019 in Wuhan, China, the COVID-19 pandemic has caused great morbidity and mortality, along with great economic loss. At the time of writing, it has infected around 22 million people and killed around 1 million globally. However, the proportion of severe disease in India has been reported to be lower (2.8%) than in other countries (15.7-29.0%) [2-6]. Reasons cited include a younger population, recurrent viral infections, environmental factors, and childhood vaccinations, including Bacille Calmette-Guérin (BCG). Epidemiological studies on the protective effect of BCG have yielded conflicting results. On one hand, it has been hypothesized that BCG vaccination may augment cellular immune response and lead to faster virologic clearance and less severe infection. On the other hand, there was no significant difference in COVID-19 incidence or outcomes in a large population-based study, which compared adults born before or after the cessation of the universal BCG vaccination program [7,8]. The relationship between COVID-19 and both active and latent tuberculosis infection (LTBI) appears to be complex and has not been adequately studied. Epidemiologic studies have shown significantly lower COVID-19 incidence and somewhat lower case-fatality rates in high tuberculosis (TB) burden countries compared to those with low TB burden [9]. However, TB was associated with severe disease and higher mortality in a recent case series from China [10]. Another conundrum is the impact of latent TB on COVID-19. Similar to BCG, it is plausible that LTBI may lead to enhanced non-specific immune response and protect against severe COVID-19 infection. However, there is no literature supporting this hypothesis. On the contrary, a small case series highlighted complications related to severe COVID-19 in three patients with LTBI [11]. Additionally, in TB-endemic countries such as India, a workup for LTBI is important in patients with severe disease as systemic corticosteroids and immunomodulators such as tocilizumab may cause reactivation of TB in this subset. As per the latest national guidelines, all patients with COVID-19 have been advised to test for TB and vice versa [12]. Therefore, the goal of this study is to evaluate LTBI in admitted COVID-19 patients and correlate it with disease severity, laboratory parameters, and outcomes.

## Materials and methods

Our hospital is a tertiary care center with a dedicated COVID-19 facility, including an isolation ward, general wards, and intensive care unit. After national directives [12], we started screening COVID-19 patients admitted to our facility for TB, including workup for LTBI. As a part of an ethically approved prospective observational study of admitted COVID-19 patients, we analyzed data for patients admitted to our facility who had been evaluated for TB. The study was approved by the Institute Ethics Committee (IEC-373/08.05.2020) and performed in accordance with the principles of the Declaration of Helsinki. Written informed consent was obtained from all patients for the observational study. As per institutional protocol, all patients having a positive Mantoux test underwent an evaluation to rule out active TB, including clinical assessment, chest radiograph, and sputum acid-fast bacilli test (if producing sputum); bedside ultrasound was used to screen for evidence of pleural effusion or lymphadenopathy. Computerized tomography was performed if indicated. The Mantoux test was administered using five tuberculin units of purified protein derivative (PPD) and was read after 48 hours [13]. Mantoux induration of 10 mm was considered positive for all individuals; for immunocompromised patients, a cut-off of 5 mm was considered positive [14]. LTBI was defined as Mantoux positivity combined with a negative workup for active TB. We scored X-rays using a simple score ranging from zero to six, with each zone marked as zero if no involvement or one if any lesion was present in that specific zone. Scoring was done independently by two fellows who were blinded to TB workup and not involved in patient management. We prospectively collected data of these patients at our center after obtaining written informed consent, as a part of an ongoing observational study. Data collected included demographic profile, symptomatology, comorbidities, blood investigations, chest X-ray, other imaging findings, duration of oxygen use, if any, duration of hospital stay, disease severity, and outcomes. Severe disease was classified as room air oxygen saturation (SpO_2_) of less than 90%, supplemental oxygen requirement, requirement of high-flow nasal cannula, non-invasive ventilation or mechanical ventilation, septic shock, or intensive care unit admission. Blood investigations, including routine complete blood counts, renal and liver functions, and coagulation, and inflammatory markers, including serum C-reactive protein (CRP) and ferritin, were recorded. Hospital discharge was as per institutional guidelines; non-severe patients were discharged once clinically stable, afebrile, and with resting SpO_2_ of more than 94% on room air, without the need for repeat negative reverse transcriptase-polymerase chain reaction (RTPCR); as per the national policy, patients with non-severe disease could be discharged 10 days after COVID-19 diagnosis, if fulfilling the aforementioned clinical criteria. However, patients without the availability of home isolation needed to be admitted until 17 days post-diagnosis. Patients with severe disease (including immunocompromised patients) were discharged once they fulfilled clinical discharge criteria and negative RTPCR reports. We studied the proportion of patients with latent TB in this cohort who underwent workup for TB. Baseline characteristics, available laboratory parameters, disease severity, radiographic extent, duration of oxygen supplementation, and hospital stay were compared between the two groups.

Statistical analysis

Discrete variables were expressed as counts (percentages) and continuous variables as mean (±standard deviation, SD) or median (minimum to maximum range). Comparability of groups was analyzed by the chi-square test, Student’s t-test, or Mann-Whitney test, as appropriate. All tests were two-tailed, and p-values of <0.05 were defined as significant. Data were analyzed using statistical software SPSS version 25.0 (IBM Corp., Armonk, NY, USA).

## Results

Initial analysis included 67 admitted COVID-19 patients who were worked up for TB in our facility during the month of September 2020. In total, 60 patients were included for analysis, as seven were found to have active TB and were excluded from the study. The mean age of the patients was 45.9 (±15.2) years and the majority (83.3%) were males. The vast majority (n = 56/60; 93.3%) had been vaccinated with single-dose BCG in infancy or early childhood. Diabetes mellitus (n = 9; 15%) was the most common comorbidity, followed by hypertension (n = 7; 11.7%) and immunocompromised status (n = 6; 10%). In addition, four (6.7%) patients had a history of previously treated extrapulmonary TB. In terms of COVID-19 severity, 35 (58.3%) patients had non-severe disease, whereas 25 (41.7%) had severe disease. Table 1 delineates the demographics, disease severity, baseline laboratory, and radiologic parameters of the included patients.

**Table 1:** Baseline characteristics of COVID-19 patients included in the study (n = 60). Data represented as mean ± SD for normally distributed data and as median (range) for non-normally distributed data; *includes active solid organ malignancy, hematologic malignancies, and HIV. COVID-19: coronavirus disease 2019; SD: standard deviation; HIV: human immunodeficiency virus

Parameters	Values
Age, years*	45.9 ± 15.2
Sex, n (%)	
Male	53 (88.3%)
Female	7 (11.2%)
History of antitubercular therapy, n (%)	4 (6.7%)
Comorbidities, n (%)	
Number of patients with comorbidities	24 (40%)
Diabetes	15 (25%)
Hypertension	7 (11.7%)
Coronary artery disease	1 (1.7%)
Chronic kidney disease	1 (1.7%)
Cerebrovascular disease	1 (1.7%)
Hypothyroidism	1 (1.7%)
Post-tubercular pleuroparenchymal fibrosis	1 (1.7%)
Immunocompromised*	6 (10%)
Severe disease, n (%)	25 (41.7%)
Laboratory parameters	
Hemoglobin (g/dl)	12.6 ± 1.9
Total leukocyte count (per mL)	5,855 (4,417.5–8,837.5)
Absolute neutrophil count (per mL)	3,560 (2,706.5–6,275.5)
Neutrophil percentage	67.5 (60.2–76.4)
Lymphocyte count (per mL)	1,100 (655.5–1,544.0)
Lymphocyte percentage	19.2 (12.0–29.7)
Neutrophil-to-lymphocyte ratio	3.6 (2.0–6.4)
Absolute monocyte count (per mL)	385 (228.5–510.0)
Monocyte percentage	7 (5.2–8.1)
Lymphocyte-to-monocyte ratio	2.8 (2.1–5.3)
Ferritin (mg/dL)	524 (121.5–1,092.5)
C-reactive protein, CRP (mg/dL)	5.2 (0.6–11.6)
D-dimer (ng/mL)	229 (86.5–383.5)
Fibrinogen (mg/dL)	466.0 (327.5–545.3)
Urea (mg/dL)	20.7 (15.0–26.0)
Creatinine (mg/dL)	0.8 (0.6–0.9)
Aspartate transaminase, AST (U/L)	47 (31–71)
Alanine transaminase, ALT (U/L)	42.8 (30.8–80.8)
Serum albumin (g/dL)	3.9 (3.4–4.3)
Hospital duration (days)	14 (11.2–18.0)
Chest X-ray score	3 (1–4)

Overall, LTBI was diagnosed in 15 (25%) of the included patients. There was no significant difference in baseline characteristics, including age, sex, comorbidities, and history of TB treatment, between patients with and without LTBI. Surprisingly, out of 25 patients with severe COVID-19, only one (4%) patient had LTBI, whereas 14 (40%) patients had LTBI among those with non-severe disease. Patients with LTBI had lower percentage neutrophil count, higher lymphocyte percentage, higher absolute monocyte count, and lower neutrophil-lymphocyte (NL) ratio compared to those without LTBI; all of these findings were statistically significant. In addition, patients with LTBI had significantly lower alanine aminotransferase (ALT) and CRP as well as lesser radiologic involvement (i.e., lower chest X-ray score). Differences between various clinical, laboratory, and radiologic parameters, as well as clinical outcomes, are delineated in Table 2. As there was no mortality, the differences could not be studied.

**Table 2:** Comparison of various parameters with respect to Mantoux positivity in included COVID-19 patients (n = 60). Data are represented as mean ± SD for normally distributed data and as median (range) for non-normally distributed data; *includes active solid organ malignancy, hematologic malignancies, and HIV. COVID-19: coronavirus disease 2019; SD: standard deviation; HIV: human immunodeficiency virus

Parameters	Mantoux positive (n = 15)	Mantoux negative (n = 45)	P-value
Age, years	48.2 ± 16.2	45.24 ± 14.93	0.5
Sex, n (%)			
Male	14 (93.3%)	39 (86.7%)	0.5
Female	1 (6.7%)	6 (13.3%)	
Past history of antitubercular treatment, n (%)	1 (6.7%)	3 (6.7%)	0.9
Diabetes mellitus, n (%)	0 (0%)	15 (33.3)	0.1
Immunocompromised,* n (%)	3 (0.2)	12 (26.7)	0.2
COVID-19 severity, n (%)			
Non-severe disease	14 (93.3%)	21 (46.7%)	0.01
Severe disease	1 (6.7%)	24 (53.3%)	
Laboratory parameters			
Hemoglobin (g/dL)	12.7 (8.3–15.5)	12.8 (7.6–16.3)	0.7
Total leukocyte count (per mL)	6,680.0 (4,180–10,740)	5,840.0 (320.0–16,790.0)	0.7
Platelet count (10^5 ^per mL)	2.4 (0.5–5.9)	2.2 (0.2–5.1)	0.4
Absolute neutrophil count (per mL)	3,516.0 (1,112.0–7,926.0)	3,831.0 (69.0–12,849.0)	0.6
Neutrophil percentage	61.0 (26.0–77.9)	69.8 (21.0–93.8)	0.03
Absolute lymphocyte count (per mL)	1,540.0 (570.0–2,892.0)	874.0 (228.0–3,235.0)	0.001
Lymphocyte percentage	26.7 (12.0–55.0)	17.7 (3.8–71.3)	0.03
Neutrophil-to-lymphocyte ratio	2.4 (0.5–6.2)	3.8 (0.3–24.7)	0.03
Monocyte count (per mL)	476.0 (259.0–730.0)	336.0 (6.0–1,200.0)	0.03
Monocyte percentage	7.5 (3.3–11.6)	6.9 (1.3–14.2)	0.2
Lymphocyte-to-monocyte ratio	4.2 (1.4–5.9)	2.6 (0.7–38.0)	0.3
Ferritin (mg/dL)	125.0 (35.3–557.0)	804.0 (32.5–1,650.0)	0.06
C-reactive protein, CRP (mg/dL)	1.2 (0.1–10.8)	6.6 (0.1–20.1)	0.02
D-dimer (ng/mL)	104.5 (37–1,194)	264 (33–774)	0.06
Fibrinogen (mg/dL)	384.5 (288–509)	493.5 (93.0–670.0)	0.07
Urea (mg/dL)	23.0 (8.0–49.2)	19.0 (7.0–151.0)	0.5
Creatinine (mg/dL)	0.6 (0.4–1.2)	0.8 (0.3–3.7)	0.2
Aspartate transaminase, AST (U/L)	37.5 (23–70)	49 (16–195)	0.2
Alanine transaminase, ALT (U/L)	38.0 (11.0–80.0)	50.0 (12.0–319.0)	0.05
Serum albumin (g/dL)	4 (3.1–4.7)	3.7 (2.7–4.9)	0.4
Overall hospital duration (days)	13 (60–36)	15 (4–54)	0.2
Hospital stay (days) (excluding immunocompromised* cases)	12 (6–17)	15 (4–30)	0.03
Duration of oxygen use (days)	0.01 (0.01–4.0)	3 (0.01–29.0)	0.002
Chest X-ray score	0.01 (0–4)	4 (0–6)	<0.001

While performing multivariate analysis, none of the factors, including raised total leukocyte count (odds ratio [OR] = 1.00; 95% confidence interval [CI] = 0.99-1.00), raised absolute neutrophil count (OR = 0.99; 95% CI = 0.99-1.00), lower lymphocyte count (OR = 0.99; 95% CI = 0.99-1.00) and Mantoux negativity (OR = 9.33; 95% = CI 0.93-93.0) could be identified as independent risk factors for severe COVID-19. In view of the small sample size and lower observations, all the inflammatory markers could not be studied in the multivariate analysis.

In addition, we compared various parameters in the studied patients with mild COVID-19, with respect to Mantoux positivity. Similar to the overall cohort, there was no significant difference in baseline characteristics and comorbidities in this subgroup. However, patients with LTBI had significantly higher lymphocyte and monocyte counts and lower radiologic involvement (chest X-ray scores) compared to patients without LTBI (Table 3).

**Table 3:** Comparison of various parameters between mild COVID-19 patients with respect to LTBI status. Data are represented as mean ± SD for normally distributed data and as median (range) for non-normally distributed data. COVID-19: coronavirus disease 2019; LTBI: latent tuberculosis infection; SD: standard deviation

Parameters	LTBI (n = 14)	No LTBI (n = 21)	P-value
Age	47.7 ± 16.7	38.9 ± 15.9	0.1
Sex (Male:Female)	13:1	17:4	0.9
Diabetes	0 (0%)	5 (23.8%)	0.06
Immunocompromised	3 (21.4%)	3 (14.3%)	0.7
Hemoglobin (g/dL)	12.3 (±2.2)	13.1 (±2.2)	0.3
Hematocrit (%)	39.1 ± 6.2	40.4 ± 6.8	0.6
Total leucocyte count (per mL)	6,925 (4,180–10,740)	4,880 (330–13,440)	0.06
Platelet count (10^5 ^per mL)	2.2 (0.5–5.9)	1.9 (0.2–4.0)	0.3
Absolute neutrophil count (per ml)	3,437 (1,112–7,926)	3,025 (69–11,070)	0.4
Neutrophil percentage	60.2 (26.0–77.9)	65.2 (21.0–82.4)	0.5
Lymphocyte count (per mL)	1,628 (993–2,892)	1,016 (228–2,406)	0.003
Lymphocyte percentage	29.4 (14.1–55.0)	22.2 (7.8–71.3)	0.6
Neutrophil-to-lymphocyte ratio	2.1 (0.5–5.2)	2.9 (0.3–10.6)	0.4
Monocyte count (per mL)	491.0 (259.0–730.0)	313.5 (6–720)	0.03
Monocyte percentage	7.2 (3.3–11.6)	7.1 (2.1–14.2)	0.6
Lymphocyte-to-monocyte ratio	4.5 (2.0–5.9)	3.5 (1.9–38.0)	0.9
Ferritin (mg/dL)	121.5 (35.3–557.0)	502.0 (32.5–1,568.0)	0.3
C-reactive protein, CRP (mg/dL)	1.2 (0.1–10.8)	0.7 (0.01–14.3)	0.9
D-dimer (ng/mL)	104.5 (37.0–1,194.0)	132.5 (33.0–519.0)	0.8
Fibrinogen (mg/dL)	365.0 (288–509)	340.5 (93–582)	0.7
Total protein (g/dL)	6.8 (5.9–7.9)	6.7 (4.5–7.7)	0.2
Serum albumin (g/dL)	4.1 (3.1–4.7)	4.1 (2.7–4.9)	0.9
Serum globulin (g/dL)	2.6 (1.8–3.8)	2.5 (1.8–3.1)	0.2
Albumin-globulin ratio	1.5 (0.5–3.3)	1.5 (0.9–2.1)	0.4
Hospital duration (days)	13 (6–36)	12 (4–54)	0.8
Chest X-ray score	0.01 (0–5)	3 (0–5)	0.03

## Discussion

This study addresses an important yet unanswered question of the impact of LTBI on COVID-19. The results indicate a potential association between LTBI and milder disease at presentation, higher lymphocyte counts, lower inflammatory markers, and lesser lung involvement, that is, lower chest X-ray scores.

Patients with LTBI-COVID-19 co-infection had higher lymphocyte and monocyte counts, along with lower NL ratio, CRP, and ALT levels. The finding of higher lymphocyte and monocyte counts was also observed in the mild COVID-19-LTBI subgroup. All these laboratory parameters have been shown to be associated with non-severe presentation and better outcomes in COVID-19 [15-17].

Our study has findings that are different from those reported so far in the literature. We found LTBI with Mantoux positivity in 25% cases, with significantly high positivity in non-severe COVID-19 compared to those with severe disease (4% vs. 40%; p = 0.01). However, in a recent in-press study from China involving 36 COVID-19 patients, 13 (36%) had interferon-gamma release assay (IGRA) positivity. IGRA positivity was associated with COVID-19 severity (p = 0.005) and rate of disease progression. Small sample size, the inclusion of active as well as post-TB sequelae cases, and the use of IGRA for diagnosing LTBI may be possible reasons for differences in results between this study and our study [10]. In another case series, the authors worked up five out of 55 severe COVID-19 patients for TB using IGRA and acid-fast bacilli sputum smears and cultures; three of these patients had IGRA positivity. One case had LTBI, another had active TB, and the third had post-TB sequelae; all three had severe disease. Two of these patients had complications; one had fungal co-infection and pneumomediastinum, and another had significant hemoptysis. The authors concluded that LTBI or active TB could lead to an increased risk of severe COVID-19 and complications during hospital stay [11]. Again, the small sample size in this study precludes a definitive conclusion on the subject of COVID-19-LTBI interaction.

Active TB, especially pulmonary involvement, can present as community-acquired pneumonia and acute respiratory distress syndrome [18,19]. This is especially possible in TB-endemic countries and immunocompromised patients such as those with HIV. Therefore, active TB can lead to poorer outcomes in COVID-19 according to some studies [20,21] and a generally benign course according to others [22,23] likely due to confounders such as advanced age and comorbidities. However, LTBI is a different scenario. It is plausible that LTBI may lead to a state of immune activation and a better immune response to COVID-19 infection. Indeed, tuberculin skin test and IGRA positivity have been stated to measure “lasting tuberculosis immune responses” and not “latent tuberculosis infection” in a recent statement by The Tuberculosis Network European Trials group consensus statement [24]. There is no evidence to suggest whether endogenous tubercular antigen-related immune response may lead to better early immune response and aid COVID-19 viral clearance or prevent dysregulation. However, we can examine the effects of the BCG vaccine on COVID-19 for some cautious inferences. Limited clinical evidence points to some preventive effects of recent BCG vaccination on COVID-19. In a recent retrospective study from the Netherlands involving 430 volunteers, BCG vaccination was associated with a decrease in the incidence of sickness during the COVID-19 pandemic (adjusted OR = 0.58; p < 0.05) and lower incidence of extreme fatigue [25]. There is some supportive data from epidemiologic studies as well. One epidemiologic study cited BCG as a potential reason for the differential involvement of the COVID-19 pandemic [8]. In another epidemiologic study, a high TB burden was associated with the lower countrywide incidence of COVID-19, irrespective of BCG vaccination status. BCG vaccination status appeared to be protective against COVID-19 in low TB-burden countries [9]. It is important to note that there is no countrywide data for LTBI prevalence to perform such correlations with COVID-19 incidence and severity.

Using the Mantoux test to evaluate LTBI may lead to false positivity with prior BCG vaccination and environmental mycobacterial infection. However, as BCG has been observed to have little impact on Mantoux positivity beyond five years of age, and the mean age of our patients was more than 45 years, BCG is less likely to confound our observations [26]. Moreover, the recent World Health Organization guidelines recommended that BCG vaccination not be considered a factor while deciding testing strategy for LTBI, in view of its limited effect on tuberculosis skin test [27]. Another potential confounder is anergy in severe viral infections [13]. However, we had similar observations in our study patients with mild COVID-19; those with LTBI had significantly higher lymphocyte counts and lesser radiologic involvement compared to those without LTBI.

This study has several strengths. This is one of the first studies to demonstrate the prevalence of LTBI among patients with varying COVID-19 severity and its correlation with laboratory parameters and clinical outcomes. Patient information was prospectively collected as a part of an ethically approved protocol. All patients were evaluated for TB by the treating team based on clinical suspicion, and a significant proportion of patients with active TB were excluded from the analysis. Baseline characteristics of patients with and without latent TB were not significantly different. We utilized PPD, a readily available, cost-effective diagnostic tool, for evaluation. Basic laboratory parameters, chest radiographs, hospital course, and outcomes were available for all patients for analysis.

However, there are several important limitations to the study. The sample size was modest. Even though the data were collected as part of a prospective observational study, the lack of recruitment of consecutive patients lends itself to selection bias. Due to the non-availability of IGRAs, only PPD was used for testing; the difference in negativity could be partially explained by anergy, as observed in severe cases of disseminated TB. The timing of Mantoux administration was not standardized, although all patients had received PPD within five days of admission. Only chest X-rays and bedside ultrasound were utilized in most cases. Advanced imaging modalities such as computed tomography were not routinely performed in all cases due to logistic limitations. There was no sample size calculation as data were analyzed as part of an ongoing observational study. Similarly, patients with mild disease were less likely to be tested for inflammatory markers such as interleukin-6, which limited our analysis of the same. No COVID-19 virologic data were available for comparison. Additionally, as patients with mild disease did not undergo RTPCR testing prior to discharge as per the national policy, time to RTPCR conversion could not be compared. There was no mortality, so the differences could not be studied. Also, as the vast majority had pediatric BCG vaccination, differences in outcomes with respect to BCG vaccination could not be assessed. Lastly, no long-term follow-up data were available to evaluate the impact of COVID-19 on TB reactivation.

## Conclusions

LTBI, as estimated using the Mantoux test, appears to be significantly less common in patients presenting with severe COVID-19 compared to non-severe disease. Patients with LTBI have significantly higher lymphocyte counts, lower levels of inflammatory markers, and extent of radiologic involvement compared to those without LTBI. Well-designed prospective studies with larger sample sizes are required to evaluate this observation.
